# Distinct responses from bacterial, archaeal and fungal streambed communities to severe hydrological disturbances

**DOI:** 10.1038/s41598-019-49832-4

**Published:** 2019-09-18

**Authors:** G. Gionchetta, A. M. Romaní, F. Oliva, J. Artigas

**Affiliations:** 1 GRECO, Institute of Aquatic Ecology, University of Girona, 17003 Girona, Spain; 2 Department of Genetics, Microbiology and Statistics, University of Barcelona, Barcelona, Spain; 3 Université Clermont Auvergne, CNRS, Laboratoire Microorganismes: Génome et Environnement, F-63000 Clermont-Ferrand, France

**Keywords:** Microbial ecology, Hydrology

## Abstract

Stream microbes that occur in the Mediterranean Basin have been shown to possess heightened sensitivity to intensified water stress attributed to climate change. Here, we investigate the effects of long-term drought (150 days), storms and rewetting (7 days) on the diversity and composition of archaea, bacteria and fungi inhabiting intermittent streambed sediment (surface and hyporheic) and buried leaves. Hydrological alterations modified the archaeal community composition more than the bacterial community composition, whereas fungi were the least affected. Throughout the experiment, archaeal communities colonizing sediments showed greater phylogenetic distances compared to those of bacteria and fungi, suggesting considerable adaptation to severe hydrological disturbances. The increase in the class abundances, such as those of Thermoplasmata within archaea and of Actinobacteria and Bacilli within bacteria, revealed signs of transitioning to a drought-favoured and soil-like community composition. Strikingly, we found that in comparison to the drying phase, water return (as sporadic storms and rewetting) led to larger shifts in the surface microbial community composition and diversity. In addition, microhabitat characteristics, such as the greater capacity of the hyporheic zone to maintain/conserve moisture, tended to modulate the ability of certain microbes (e.g., bacteria) to cope with severe hydrological disturbances.

## Introduction

Microbes inhabiting intermittent streambeds are responsible for the control and development of many biogeochemical processes essential for fluvial ecosystem functioning. Despite flow intermittency being a natural part of the hydrologic cycle of intermittent streams, the adaptation of sediment microbiota (archaea, bacteria and fungi) to alternate dry-wet series could be modified depending on the duration, intensity and frequency of the disturbance^1,2^. The hydrology of intermittent Mediterranean streams consists of irregular flow, such as low or zero flow between spring and summer and low or regular flow during autumn and winter^3^. Depending on the degree of intermittency, the flow regime can decrease or disappear, and isolated pools can form along a stream’s path.

Current global models predict temperature increases in the Mediterranean Basin, coupled with strengthened periods of drought and intense sparse flash storm episodes^4,5^. In this context, water scarcity is usual in Mediterranean freshwater ecosystems that experience the typical climate combination of high summer temperatures and extreme seasonal variation in rainfall patterns^6^. Conditional events, such as drought and rewetting, can alter resource availability and/or the physical environment, affecting the structure of a community, population or ecosystem. Based on their temporal patterns, such unforeseen events have been classified as pulse, press and ramp disturbances^1,7,8^ and may promote distinct consequences on the microbial community. Pulses are generally short-term and intense disturbances (e.g., flood, rewetting or rainfall after a long dry period); press disturbances describe conditions that worsen as the stressor persists, whereas ramps reflect disturbances whose strength increases in time without an endpoint (e.g., droughts)^1^.

Archaea, bacteria and fungi are the most important microbial groups with pivotal functions in an ecosystem. Their contribution to nutrient recycling and biogeochemical in-stream processes necessitate exploring their responses to the long-term drought disturbance for intermittent Mediterranean streams. On the one hand, studies have reported that microbial communities inhabiting temporary streambeds^2,9^ and soils^10,11^ change their functions and structure when subjected to prolonged dry conditions. However, recent research has highlighted the observed capacity of microbes to develop resistance and drought legacy in the case of repeated perturbations^12–14^. These contrasting observed responses could be due to intrinsic differences in the microbial life strategies of bacteria, archaea and fungi, and/or in the occupied streambed microhabitat (such as surface and hyporheic zone sediments or decomposing plant material accumulation). Additionally, responses may be modulated by the timing and severity of drought/rewetting episodes. In the present study, this knowledge gap is addressed by simulating different and severe climate change scenarios (i.e., long-term drought, punctual storms interrupting the drying process and abrupt rewetting), including the study of archaeal, bacterial and fungal communities and three main streambed habitats (surface and hyporheic sediments and buried leaves), to provide an additional step in predicting global change effects on streambed microbial ecology.

Focusing on the three microbial groups, the few recent studies available on archaea have reported that their presence is ubiquitous in different environments (such as inland and marine sediments, hot springs, hydrothermal vents, solfataras, salt and soda lakes) and their great contribution to ammonia oxidation in terrestrial and/or freshwater sediment habitats^15–17^. However, little is known about the diversity of archaea in inland freshwater ecosystems subjected to water stress conditions. At the same time, fungi (especially in dry sediment) are generally recognized as the most resistant group to drought, owing to their specific molecular and physiological traits (i.e., thicker and hydrophobic cell walls, mycelial growth, and spore motility, among others), which facilitate their adaptation to arid systems^18,19^. Regarding bacteria, drought effects could lead to differing responses that mainly depend on habitat characteristics (such as sediment moisture, organic matter content and oxygen level), hydrology history and drought duration and finally on the specific phylum being considered^2,20,21^. Despite being the most studied microbes in temporary streams, knowledge on bacterial responses under prolonged streambed desiccation is still limited^22–24^. According to the National Oceanic and Atmospheric Administration^25^, a prolonged drought is defined as a dry period lasting more than three months. Such a period may transform temporary aquatic ecosystems into terrestrial systems, reducing the flow zones (usually in upstream branches) and increasing sediment heterogeneity and patchiness, which results in the formation of distinct microhabitats within the streambed. Therefore, the specific habitat where microbial communities develop is a key point that may determine their resistance and response to extreme hydrological intermittency. Greater pore-scale heterogeneity and aerobic or anaerobic microhabitats would promote the spread of highly diverse known and rare taxa^26^. Free niches such as anoxic pools, oxygenated dry sediment, humid hyporheic layers and buried leaf packs may induce the selection of certain microbial groups and opportunistic taxa that display physiological acclimation strategies^27–29^. Furthermore, the occurrence of sporadic storm events in the dry streambed may further modify the physical and chemical conditions of the streambed habitat (water, organic matter, and availability of nutrients) with consequences for microbial functioning^30^ and community composition. Thus, the variety of micro-habitats and storm episodes would further differentiate the microbial response trajectories, already recognized as multifaceted, between dry and wet phases.

Accordingly, the main objective of this study was to report on how microbial communities present in different sediment microhabitats (surface, hyporheic, and buried leaves) cope with the extreme alteration in hydrological intermittency in freshwater ecosystems. Specifically, we assessed how archaeal, bacterial and fungal community structure and composition were affected by (i) hydrological treatment effects, (ii) treatment effects specific to microhabitat and (iii) treatment effects in terms of taxonomic class abundance variations over time.

Microbial communities were sampled from sediment microcosms subjected to three treatments under controlled light and temperature conditions. After five months of drought simulation, all microcosms were subject to a one-week rewetting phase, simulating stream flow recovery and water table resumption. We expected that microbes inhabiting the surface sediment (as opposed to the other streambed habitats, i.e., the hyporheic zone and buried leaves) would respond more strongly to long-term drought due to the more direct exposure to desiccation effects. The moisture of the hyporheic zone and that of buried leaves are expected to buffer desiccation effects on microbes occupying these habitats. In addition, among the microbial communities, we hypothesized that given the specific molecular traits of fungi and the archaeal capacity of inhabiting extreme environments^19,31,32^, fungi and archaea would be the most resistant groups to drought.

## Results

### Initial microbial communities

The taxonomic composition of the initial bacterial, archaeal and fungal communities showed no significant differences between treatments (Table S1, Fig. S1). In terms of phylum and class composition, the initial bacterial communities were dominated by Proteobacteria (50% relative abundance in sediments and approximately 65% in leaves, Fig. S1A). Within the Proteobacteria group, Alphaproteobacteria was among the most abundant classes in both types of sediment (20% relative abundance), while Gammaproteobacteria was among the most abundant in leaves (30% relative abundance, data not shown). In the archaeal communities, Thaumarchaeota was the dominant phylum (75% relative abundance in the surface and hyporheic sediments, Fig. S1B). Within the Thaumarchaeota phylum, the Nitrosopumilales and Nitrososphaerales classes were the most abundant (44% and 34% relative abundance, respectively, data not shown). Finally, Ascomycota, Basidiomycota and Cryptomycota were the most dominant fungal phyla in the sediments with high phyla and class variability between treatments in the surface and hyporheic sediments (Fig. S1C). The fungal community inhabiting the leaves was dominated by Ascomycota (80% relative abundance, Fig. S1C) and Dothideomycetes classes (78% relative abundance, data not shown). On the other hand, the initial diversity calculated as Shannon-Wiener and richness indices was higher, as absolute values, for bacteria compared to that of archaea and fungi (Table S2).

### Responses of microbial community structure to treatments across habitats

The structure of the microbial communities was shaped by the treatments, but the magnitude of these changes mainly depended on the type of streambed habitat considered and the experimental time (surface and hyporheic sediments or buried leaves, Table 1, Table S2). In the sediments, bacterial richness and diversity were affected by the treatments interactions with time and by the habitat factor, with decreasing values occurring at the surface in the Dry (D) treatment at the end of the long-term drought and beginning of rewetting (Table 1, t150-t151 in Table S2). In the leaves, bacterial diversity and richness were also reduced by the treatments, especially in the D community at the end of the long dry phase (Tables 1 and S2). Archaeal richness and diversity were affected by the treatments and habitat factors, where richness values decreased in the Dry (D) and Dry-Storms (DS) treatments of the surface sediment, and those of the hyporheic sediment were more consistent during the experimental drying time (Tables 1 and S2). Fungal richness and diversity were the least affected, and only the fungal diversity of the hyporheic Control (C) and Dry (D) treatments was reduced when the sediments were rewetted (Tables 1 and S2). The percentages of shared OTUs between treatments were equal to averaged values of 7.21%, 6.87% and 0%; and 8.67%, 6.23% and 0% for surface and hyporheic sediments and bacteria, archaea and fungi, respectively (Fig. S2). In the buried leaves, the percentage of shared OTUs between treatments was on average 7.05% and 3.75% for bacteria and fungi, respectively (Fig. S2). The greatest time variation in shared OTUs was observed for archaea in surface sediments, which showed a gradual decrease during drought and an increase during rewetting (Fig. S2). Shared OTUs in bacteria inhabiting surface sediment followed a similar but less marked trend to that of archaea (Fig. S2). In the hyporheic sediment, the percentage of shared OTUs was more constant over time, while in the buried leaves, this percentage decreased by the end of the long-term drought and did not recover during the wet phase (Fig. S2).

**Table 1:** Significance of PERMANOVA results (p-values) for Shannon-Wiener (H) and richness (S) indices (raw values are presented in Table S2) and for the phylogenetic Unifrac distance.

A.	SHANNON (H)			RICHNESS (S)			UNIFRAC		
	Bacteria	Archaea	Fungi	Bacteria	Archaea	Fungi	Bacteria	Archaea	Fungi
TR	*0.061*	**0.029**	**0.044**	0.245	**0.005**	0.425	**0.001**	0.443	0.261
TI	**0.002**	0.227	*0.091*	**<0.001**	0.486	0.128	0.265	0.231	0.907
HAB	**0.004**	**0.017**	**0.052**	**0.002**	**0.049**	0.223	**0.001**	**0.009**	0.302
TRxTI	**0.043**	0.146	0.759	**0.002**	0.672	0.289	0.540	0.451	0.778
TRxHAB	0.196	0.778	*0.070*	**0.004**	0.221	0.131	**0.002**	**0.056**	**0.037**
TIxHAB	*0.098*	0.065	0.262	**0.004**	0.110	0.273	0.952	0.180	0.778
**B**.	**SHANNON (H)**			**RICHNESS (S)**			**UNIFRAC**		
**SUR**	**Bacteria**	**Archaea**	**Fungi**	**Bacteria**	**Archaea**	**Fungi**	**Bacteria**	**Archaea**	**Fungi**
TR	0.310	0.472	0.110	0.412	0.170	0.347	**0.001**	0.113	0.541
TI	*0.073*	0.421	0.188	**0.005**	0.196	0.365	0.781	0.193	0.918
**HYP**	**Bacteria**	**Archaea**	**Fungi**	**Bacteria**	**Archaea**	**Fungi**	**Bacteria**	**Archaea**	**Fungi**
TR	0.250	**0.044**	**0.010**	0.145	**0.002**	0.366	0.717	0.176	**0.001**
TI	**0.050**	0.075	**0.042**	**0.013**	0.163	0.246	**0.038**	0.184	**0.034**
**LEAVES**	**Bacteria**	**Archaea**	**Fungi**	**Bacteria**	**Archaea**	**Fungi**	**Bacteria**	**Archaea**	**Fungi**
TR	**0.004**	*na*	0.470	**0.007**	*na*	0.838	0.971	*na*	0.339
TI	0.170	*na*	0.348	**0.039**	*na*	*0.096*	**0.033**	*na*	0.347

The diversity and distance matrices were calculated from the OTU table of bacterial, archaeal and fungal assemblages inhabiting three distinct habitats, indicated as SUR, surface sediment; HYP, hyporheic sediment; and LEAVES, buried leaves. (A) Results from PERMANOVA models including three factors (treatment, TR; time, TI; and habitat, HAB). Here, the *Habitat* factor includes surface and hyporheic sediment habitats (and not buried leaves) since the sampling time sequence was shorter for the leaves. (B) Results from PERMANOVA considering each habitat individually and including two factors (treatment, TR and time, TI). Significant differences (p < 0.05) are indicated in bold, while those at the limit of significance (p < 0.1) are indicated in italics; the *na* stated for data not available.

### Responses of microbial community composition to treatments across habitats

**Figure 1:**
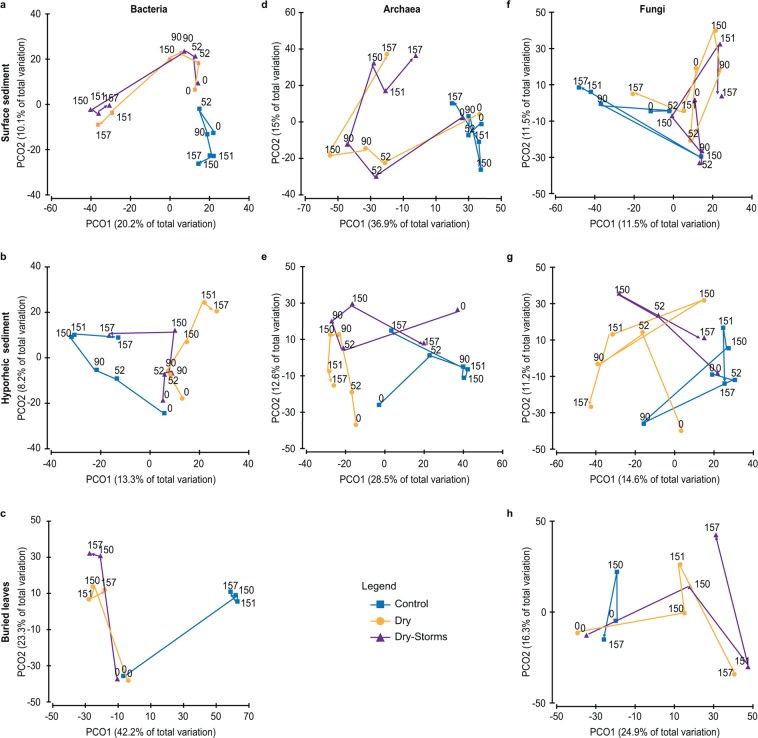
PCO from bacterial, archaeal and fungal OTUs inhabiting the three habitats, indicated as surface sediment, hyporheic sediment, and buried leaves. The arrows indicate the temporal evolution. The colour pattern is indicated in the legend for the three treatments: Control (C); Dry (D); Dry-Storms (DS).

In terms of composition, the bacterial and archaeal communities showed significant differences between treatments and types of habitat, and both factors interacted (Fig. S3, Table S3). The fungal communities also responded significantly to the types of streambed microhabitats and to their interactions with the treatments (Fig. S3, Table S3). To better highlight the effects of the treatments, further analyses for each habitat (surface and hyporheic sediments or buried leaves) were independently carried out (Fig. 1, Table 2).

**Table 2:** Resulting *p*-values from the PERMANOVA analyses assessed for each microbial community (bacteria, archaea, and fungi) inhabiting the three habitats: surface sediment, hyporheic sediment and buried leaves.

SURFACE SED.	Bacteria	Archaea	Fungi
*Treatments*	**0.004**	**0.014**	**0.005**
*C, D*	**0.002**	**0.021**	**0.026**
*C, DS*	**0.002**	**0.004**	**0.016**
*D, DS*	0.438	0.814	0.351
**HYPORHEIC SED**.	**Bacteria**	**Archaea**	**Fungi**
*Treatment*	**0.001**	**0.003**	0.197
*C, D*	**0.002**	**0.003**	—
*C, DS*	**0.016**	*0.063*	—
*D, DS*	*0.082*	**0.035**	—
**BURIED LEAVES**	**Bacteria**	**Archaea**	**Fungi**
*Treatment*	**0.018**	n.a.	0.667
*C, D*	**0.030**	n.a.	—
*C, DS*	*0.067*	n.a.	—
*D, DS*	0.506	n.a.	—

Significant differences (p < 0.05) between *treatments* (C, Control; D, Dry; DS, Dry-Storms) and relative pairwise comparisons are indicated in bold, while those at the limit of significance (p < 0.1) are indicated in italics.

The bacterial communities inhabiting the surface and hyporheic sediments in the D and DS treatments were significantly different from those in the C treatment (Fig. 1a,b, Table 2). Furthermore, the bacterial communities colonizing the leaves buried in the dry sediments (D and DS treatments) were significantly different from those found in the C treatment (Fig. 1c, Table 2; at the limit of significance in the case of DS, p = 0.06). For archaea, the surface sediment communities in the D and DS treatments were significantly different from those in the C treatment (Fig. 1d, Table 2).

**Figure 2:**
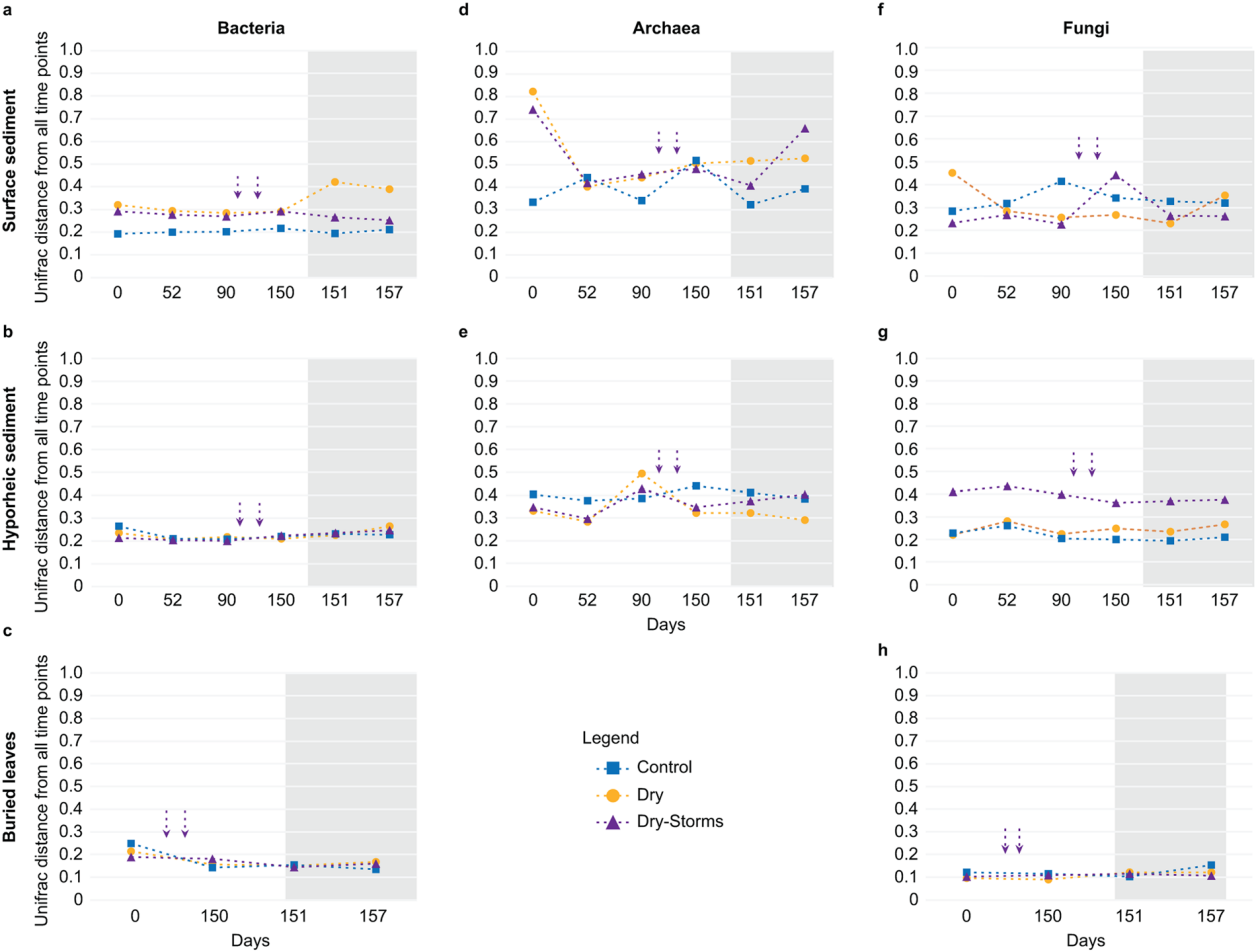
Time changes in the average weighted UniFrac distances between each sample and all other samples from the same treatment for bacterial, archaeal and fungal communities inhabiting surface and hyporheic sediments and buried leaves. Higher points on the y-axis indicate samples with greater phylogenetic distances with respect to the rest of the samples from the same treatment. The shaded area indicates the rewetting phase. The colour pattern is indicated in the legend for the three treatments: Control (C); Dry (D); Dry-Storms (DS). Vertical purple arrows indicate when storms occurred for the DS treatment.

Community composition trajectories during the experiment indicated that in both D and DS, the bacterial communities in the surface sediment were gradually modified during the drying process and eventually differed from those in the C community. However, the rewetting in D and the second storm in DS resulted in the largest community changes when compared to that in the C community (Fig. 1a). Although a shorter time sequence was presented, similar trajectories were observed for the bacteria in the buried leaves (Fig. 1c). In the hyporheic zone and upon rewetting, the bacterial communities inhabiting the DS returned to a community composition closer to that in C (T157, Fig. 1b), whereas the D community remained separated from C and DS (Fig. 1b). Overall, the bacteria inhabiting the surface sediment in the dry conditions (D and DS) showed greater phylogenetic distances compared to that of C throughout the experiment (Fig. 2a, Table 1B). In the case of the surface bacteria in D, phylogenetic separation was particularly evident just after rewetting (day 151, Fig. 2a). Regarding the hyporheic zone and buried leaves, bacterial communities showed lower phylogenetic distance values overall, apparently unaltered between treatments (Fig. 2b,g, Table 1B).

As in the case of bacteria, rewetting and the second storm determined significant changes in the archaeal community compositions of D and DS, respectively (Fig. 1d, Table 2). However, in contrast to the example of bacteria, in the surface sediment, the archaeal community trajectories of the D and DS communities were split from that of C after 52 days of drying (Fig. 1d, Table 2). In the hyporheic sediment, significant differences were found between C and both the D and DS archaeal communities (Fig. 1e and Table 2; at the limit of significance in the case of DS, p = 0.06). Furthermore, the hyporheic D community was significantly different from that of DS, which, at the end of the wet phase (DS day 157, Fig. 1e, Table 2), was clearly trending towards recovering its composition and being closer to C. In terms of phylogenetic distance, the archaea inhabiting the surface sediment showed higher distance values and a greater effect of treatments compared to those in the hyporheic zone (Figs 2c,d, Table 1A, Habitats and Treatment × Habitats effects) and, above all, to those of bacteria (Figs 2a,b).

The fungal communities inhabiting the surface sediment showed significant effects of treatment on their composition, whereas no effect was found in the hyporheic sediment and buried leaves (Table 2). In particular, in the surface sediment, treatment C was significantly different from either D or DS (Fig. 1f, Table 2). The phylogenetic distance of the fungal community inhabiting the surface sediment and buried leaves indicated no significant effect of time or treatment (Fig. 2e,h Table 1B). However, in the hyporheic sediment, the phylogenetic distance was significantly affected by treatment and experimental time, with the highest values being measured in the DS treatment (Fig. 2f, Table 1B).

### Variation in microbial class abundances due to treatments

**Figure 3:**
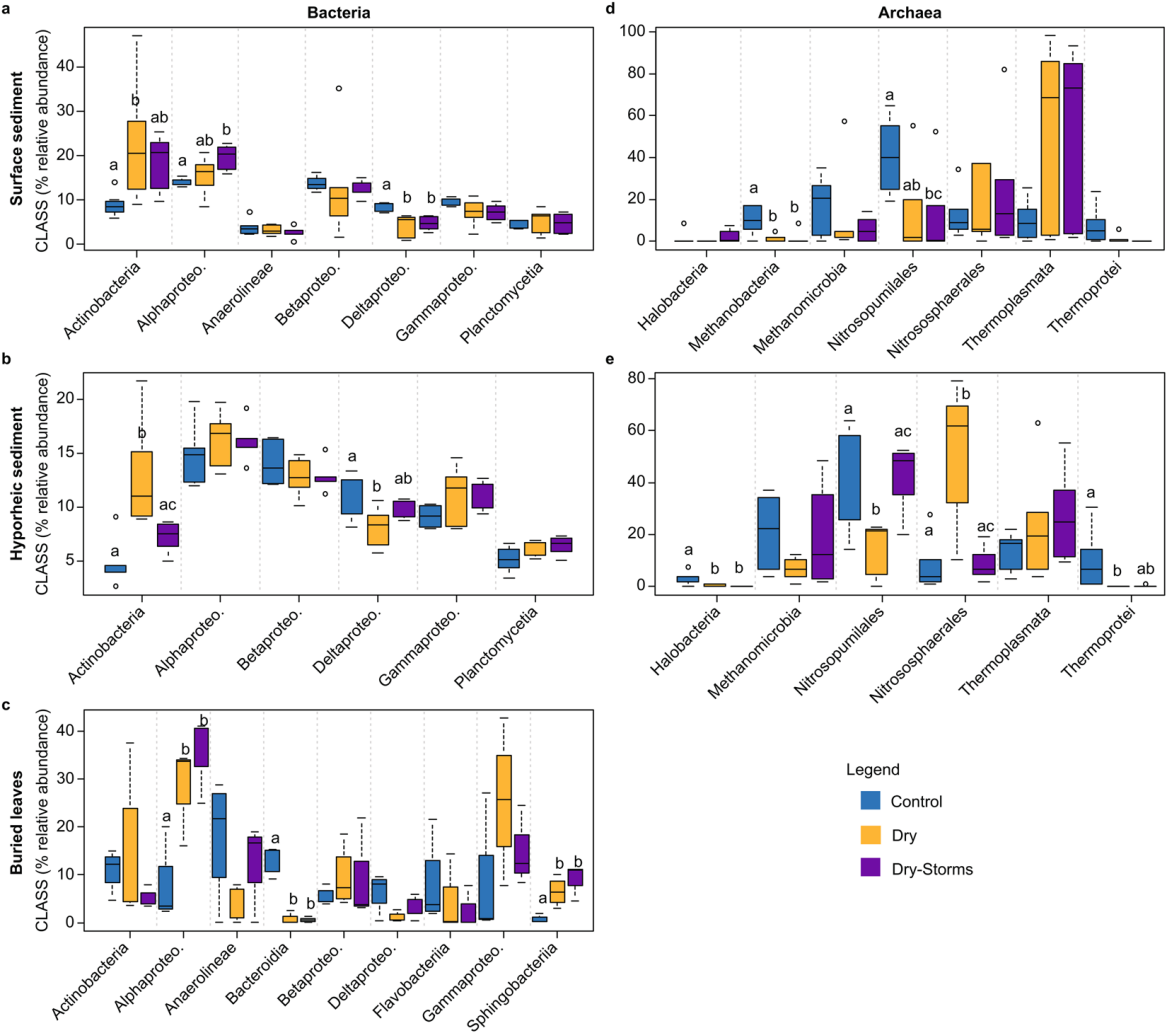
Boxplots for bacterial and archaeal class variability (percentage of relative abundance) considering only those classes reporting abundances higher than 5% throughout the entire experimental period. To better visualize where changes occurred, each axis is on a different scale of abundance. The three habitats are indicated as surface sediment, hyporheic sediment, and buried leaves. The colour pattern is indicated in the legend for the three treatments: Control (C); Dry (D); Dry-Storms (DS).

Each microbial group considered showed a specific sensitivity to drought and watering events (as storms and rewetting) in terms of taxonomic composition, although these changes also strongly depended on the habitat considered. Regarding bacteria, overall, the D and DS treatments resulted in an increase in the relative abundance of Actinobacteria and Alpha*-*proteobacteria and a decrease in Delta-proteobacteria in the surface sediments compared to those in the C treatment (Fig. 3a). Similarly, in the hyporheic sediment, the D and DS treatments increased Actinobacteria and decreased Delta-proteobacteria, with significant changes in the D treatment (Fig. 3b). In the case of the bacterial groups inhabiting the buried leaves, the Alpha-proteobacteria and Sphingobacteria classes increased significantly in D and DS, whereas Bacteroidia sharply decreased (Fig. 3c). Regarding archaea, changes at the class level in the D and DS treatments compared to those in the C treatment were observed in surface and hyporheic sediments (Fig. 3d,e). Thermoplasmata was strongly enhanced in the D and DS communities during drought, while Nitrosopumilales and Methanobacteria decreased. Finally, fungal diversity at the class level did not show any differences between treatments since high variability in community composition was already observed in the C treatment (Figs S4 and S6).

The observation of the taxonomic classes’ relative abundances revealed fluctuations over the duration of the experiment. Bacterial class abundance in the surface sediment under long-term drought conditions showed that Actinobacteria gradually increased by as much as 20% compared to that at the beginning of the experiment (at day 150 in D, Fig. S5A). Within Actinobacteria, the abundances of *Arthrobacter* and the *Nocardioides* genera (both gram-positive Actinomycetales) during the drought phase corresponded to 3% and 2%, respectively, of the total genera presented at the end of the drought phase (D at day 150, data not shown). The first 24 hours of rewetting resulted in a peak in the increase in Actinobacteria and Bacilli and in a Proteobacteria (Delta*-*, Beta*-* and Gamma*-*) reduction in the D bacterial communities inhabiting the surface sediment in D (on day 151, Fig. S5A). However, at the end of the wet phase, the proportion of class abundance returned to levels similar to the initial ones, albeit with a two-fold relative abundance of Beta-proteobacteria (D on day 157, Fig. S5A). In the DS treatment, temporal changes in the relative abundance of the classes were less evident, with an increase of Actinobacteria (6%) at the end of the drought phase (DS on day 150, Fig. S5A). In the hyporheic sediment, the proportion of the main bacterial classes was similar to that of the surface sediment, albeit with a dominance of Alpha-proteobacteria and Beta-proteobacteria, and time changes were not observed. However, overall Actinobacteria abundance tended to be greater in D compared to that in C and DS (Fig. S5B).

The abundance of the Alpha-proteobacteria class, colonizing the buried leaves under the D and DS treatments, increased approximately 34% at the end of the dry phase compared to its initial values (from time 0 to time 150, Fig. S5C). Rewetting did not cause any major changes in the composition of the bacterial communities inhabiting the hyporheic zone or the buried leaves but caused an increase in the abundance of Actinobacteria at the end of the wet phase (day 157 in D, Fig. S5B, Fig. S5C).

**Figure 4:**
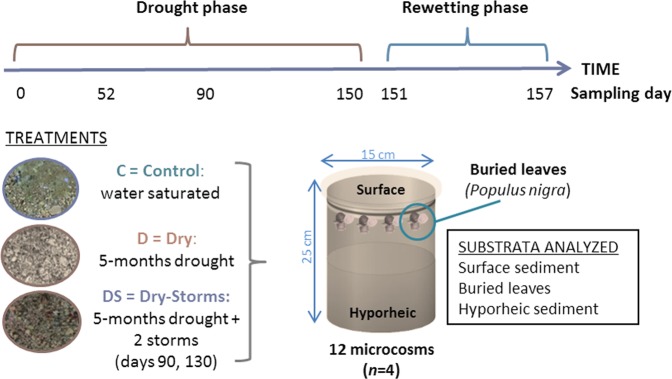
The experimental design (Photos: G. Gionchetta).

In terms of the archaea communities, the D and DS treatments experienced a reduction of Nitrosopumilales and Methanobacteria throughout the experiment in surface sediments (Figs 4D and S5D), while Nitrososphaerales decreased with the drought phase but not the wet phase (Fig. S5D). Thermoplasmata was considerably enhanced in the surface sediment, becoming dominant in the D and DS communities during the drought phase (>90%, Figs 3D and S5D). *Ferroplasma* and especially *Thermogymnomonas* were the Thermoplasmata genera that expressed the greatest increase in abundance during the long-term drought phase (data not shown). Nevertheless, *Nitrosopumilus* (Nitrososphaerales class) and *Nitrososphaera* (Nitrosopumilales class) genera were considerably reduced during the drought phase (data not shown). Shifts in the abundance of classes were also observed during rewetting. In particular, on day 157 in the D and DS surface groups, Methanomicrobia (mainly *Methanolinea* genus) and Nitrososphaerales increased their abundance by 60% and 40%, respectively, while Thermoplasmata largely decreased (<10%, D and DS at day 157, Fig. S5D).

In the hyporheic sediment in treatment D, Nitrososphaerales clearly increased during both the drought and wet phases (Figs 4E and S5E), but Nitrosopumilales significantly decreased as the experiment progressed, almost disappearing by the end of the wet phase (Fig. 3E; day 157, Fig. S5E). The relative abundance of the archaeal classes overall was steadier in the DS hyporheic sediment (Figs 3E and S5E).

The fungal communities inhabiting the buried leaves showed that Dothideomycetes and Tremellomycetes (mainly *Cystofilobasidiales* and *Tremellales* genera) increased in treatment D during the drought phase, while Leotiomycetes were favoured by the DS treatment (Figs S4 and S6). However, even by the end of the rewetting phase, the high variability of the fungal community in the leaves was still evident. Agaricomycetes was dominant in the C treatment, whereas Hymenoscyphus was dominant in D and Eurotiomycetes in DS (on day 157, Fig. S6).

## Discussion

Shifts in microbial taxonomic groups were observed under different hydrological conditions which led to larger impacts on the surface microbial community composition and diversity, especially when sporadic storms and rewetting occurred. Moreover, specific microhabitat characteristics (e.g. the moisture of hyporheic sediment) modulated the ability of certain microbes to cope with severe hydrological stress. Among the microbial groups, the archaeal community composition was the most modified and showed greater phylogenetic distances. The complexity of the experimental design, such as the frequency of sampling and the number of variables considered (time, treatment, and microhabitat) to study the three distinct microbial communities in sediment microcosms, presented the technical limitation of pooling replicates. This method required caution in terms of obtaining strong conclusions from the results and the use of proper and robust statistical tools, as those applied. It is thus advisable for future studies to sequence all the replicates available and/or simplify the experimental design to reinforce the observations.

When subjected to long-term drought, bacteria and archaea showed similar transitions to more terrestrial communities in terms of relative class abundance, but the response mechanism varied. Under different environmental and hydrological conditions, resource competition dynamics most likely determined the differences observed in the community responses. The bacterial community gradually changed from the outset without any drastic modifications observed, whereas archaea presented a larger shift in community composition, at day 52 and onward. Surprisingly, the archaeal community changed more than that of the bacteria, but both microbial groups showed increments of some drought-favoured class abundances, which were also the most present in terms of OTU sequences. Large variation in the abundance of certain classes was reported among archaeal communities. Similar to previous studies, the Thermoplasmata class (Euryarchaeota phylum) dominated the dry phase^17,33^. Within this class, the *Thermogymnomonas* genus was dominant in the surface sediment of the dry (D) treatment (88% relative abundance), which was probably due to its observed capacity to cope with osmotic and drought stress^34^. On the other hand, Nitrosopumilales and Nitrososphaerales (Thaumarchaeota phylum) presence considerably decreased under long desiccation, and this abundance response trend was in contrast to those in previous studies that identified Nitrososphaerales as dominant in many dry streams^33^. Furthermore, within this class, the *Nitrososphaera* genus, known as an important player in the nitrification of intermittent streams^35,36^, was extremely reduced, potentially leading to consequences for the streambed nitrogen cycle (i.e., reducing nitrate leaching). The discrepancies in the class abundance fluctuations observed between the field-study results in the literature and our experimental findings could be explained by an additional laboratory stress (e.g., simplified community interactions between biotic and abiotic factors due to the exclusion of environmental inputs) or could have been due to the specificity of the microbial community inhabiting the stream sediment sampled and inoculated into the columns.

For the bacteria, the long-term drought caused some changes in their composition, as shown by the increasing abundance of certain taxa, especially within the surface sediment. The spread of Actinobacteria and Firmicutes (mainly Bacilli), characteristic taxa of arid and soil environments^37–39^ observed during the drought, suggested the beginning of a terrestrial transition. Actinobacteria have already been described as being able to survive lengthy dry periods^40^ given the strong, thick and interlinked peptidoglycan cell wall of gram-positive bacteria (i.e., Actinobacteria and Firmicutes) and their capacity to form colonies that often grow extensive mycelia (like fungi) allowing the habitat to be explored, water to be sought and dormant cells appear^37,41^. More specifically, in this study, the *Arthrobacter* genus (Actinobacteria class, Actinomycetales order) were dominant in the desiccation period, which might have been facilitated by its capacity to resist desiccation and starvation^42^ and its ability to metabolize aromatic compounds as its sole carbon source^43,44^. The desiccation process combined with external inputs (e.g., leaves fall during late summer-autumn) generally increased the accumulation of lignin-like compounds in the streambed^45^; in this experiment, the exclusion of external inputs suggested that the organic matter had relatively high aromatic quality due to its persistence in the sediment where it accumulated during the desiccation period.

In contrast to the surface sediment, the bacterial community inhabiting the hyporheic sediment resisted the long-term drought better than the superficial drought, and the phylogenetic distances and the shared OTUs between the treatments were apparently less influenced by the hydrological changes throughout the experiment. The responses of fungal community composition to the experimental conditions were difficult to interpret since the fungal communities were the most variable group from the beginning of the experiment. The large variability observed at the initial time could have enhanced the diversification of the taxa present and the associated capacities to cope with disturbances. Additionally, the high number of micro-niches potentially created during the drought phase might have determined the presence of dormant spores that were germinated during the course of the experiment, and vice versa, thus increasing the variability observed in the fungal group at each experimental time. Similar to the results of previous studies, the majority of Dothideomycetes, especially those observed in the buried leaves, expressed their amphibian nature and their preference for colonizing leaf litter and plant material^46,47^. Despite the range of dynamics in the fungal communities, in the surface sediment, they were significantly affected by the long-term drought, similar to that observed for the bacteria and archaea.

The integrated nature of our findings by including all experimental phases (drought, storms and rewetting) revealed that the largest shift in community structure and composition occurred under sporadic storms or the rewetting phase, which, in turn, seemed to influence the microbial biodiversity, especially for the communities colonizing the surface sediment. Specifically, the consequences of the wet events affected the changes in the relative abundance of the classes, the reduction in OTU richness and the abrupt variation in community similarities, especially from the dry (D) bacteria and dry-storms (DS) archaeal communities.

Generally, a rewetting phase subsequent to a dry period initiates a cascade of physical and biological events^48^, resulting in enhanced heterotrophic microorganism respiration^10^, awakened dormant cells^49,50^, nutrient mineralization, the mobilization of carbon protected in aggregates (as after storm events), and the release of intracellular osmolytes^24,37,51^. In this study, the occurrence of water after a long dry period might have broken the sediment aggregates and compromised the stability achieved with the extracellular polymeric substances produced during the drought phase^30^ or caused the cell wall breakage^52^ of certain microbes that did not utilize the appropriate osmolytes to compensate for the rapid osmotic variations in the media^53^. These physicochemical consequences, combined with the gravitational water movement, might have determined the release of intact aggregates as well as the release of already dead cells stuck in the sediment and thus determined the change in the composition of the community. In parallel, the release of nutrients within the rewetted sediment might have triggered the spread of certain microbial groups metabolically adapted to the osmotically changed conditions, for instance, the archaeal Methanomicrobia, Nitrosopumilales and Nitrososphaerales inhabiting the surface sediment. These groups may, in turn, promote processes related to both carbon and nitrogen cycles^54,55^. A similar process occurred for the bacteria, where Proteobacteria (mainly Alpha- and Beta-proteobacteria) increased their abundance in the surface sediment after rewetting in the community from the drought treatment. Interestingly, these bacteria have already been identified as a group that responds positively to rewetting^10,23,56^.

On the other hand, even though the storm events seemed to modify prokaryote diversity, mainly in the surface sediment, and barely influenced their composition, hidden consequences from the precipitation events were revealed when comparing the responses of the communities from the dried sediments (Dry treatment) with those previously wetted by storms (Dry-Storms treatment) at the end of rewetting. The bacteria and archaea inhabiting the hyporheic sediments in the Dry-Storms condition returned to the relative abundance of the classes and to similar community compositions to those seen at the beginning of the study. In parallel, a comparable trend was reported in Dry-Storms surface bacteria, although the trend was evident only in terms of class abundance. These observations suggested that the simulated sporadic storms interrupting the long dry period could have promoted and boosted microbial recovery, at least in terms of class composition.

As hypothesized, the microhabitat characteristics emerged as a relevant factor that might influence microbial community composition under both water stress and rewetting situations. The surface sediment was the habitat most affected by the hydrological disturbances, followed by the buried leaves and hyporheic sediment, where the latter may buffer the hydrologic impacts up to a certain point. In comparison to archaeal communities, the bacterial communities, inhabiting the hyporheic sediment, remained almost unchanged in terms of diversity and class abundance, likely due to the combination of higher humidity and a higher fraction of fine particles^30^ and thus resource retention^57^. The higher moisture of the hyporheic zone compared to that of surface sediment (3.41% and 0.97% on average for Dry and Dry-Storms for the hyporheic and surface sediment, respectively)^30^ may have resulted in the lower effect of the long dry phase on the microbial communities, as suggested above. However, the community composition in the hyporheic zone was still significantly affected by the long-term drought, thus not fully coinciding with that in the control treatment after rewetting.

The overall findings of this study contribute to our understanding of the responses of streambed microbial communities to future hydroclimate variability. The risk of potential and irreversible modifications in the diversity and composition of intermittent streambed microbial communities under increasing climatic and anthropogenic pressures could have important consequences for the development of several in-stream sediment processes, such as biogeochemical reactions, water quality maintenance and nutrient cycles.

## Methods

### Microcosm set-up

The long-term drought experiment was performed in 12 streambed sediment microcosms randomly assigned to three treatments (n = 4 per treatment): Control (C, maintained in wet, isolated pool-like conditions), Dry (D, 5-month drought), and Dry-Storms (DS, 5-month drought, including 2 flash storms). After five months of the drought treatment, all the microcosms were subjected to one-week rewetting, simulating stream flow recovery and water table resumption (Fig. 4).

The microcosms consisted of dark plastic cylinders (PVC), the base of which had been perforated with small holes (3 mm diameter) to allow water movement along the sediment column. Each microcosm had two small windows (ca. 1 cm^2^) at a depth of 22 cm from the surface and on opposite sides of the microcosm to allow hyporheic sediment sampling. Oxygen micro-sensors (PreSens^®^ optical fiber sensor spots, Precision Sensing GmbH, Germany) were positioned on the surface and 22 cm inside the microcosm (one per treatment) to monitor dissolved oxygen variations (Table S4A). The 12 microcosms were prepared *in situ* in early February 2016 and filled with streambed sediment from the “Santa Llúcia de Puigmal” stream, a headwater tributary of the Fluvià River (42.217637N, 2.401402E) displaying a typical Mediterranean discharge pattern (summer discharge reduction and flow recovery in autumn-winter). The water used for the entire experiment was collected from the same stream site. Approximately 50–75 L of water was collected every two weeks, filtered through 0.2 μm pore size nylon filters (Whatman, Kent, UK) to ensure no microbes were added to microcosms when wet events were simulated or during the maintenance of the Control treatment and kept in fresh conditions (4–10 °C). Before use, the filtered water was acclimated to room temperature for approximately two hours. Sediment stream sampling was performed under base flow conditions, and the physicochemical parameters of the stream water were monitored to ensure these parameters were not modified during the experiment (Table S5).

Once the sediment microcosms were created and filled with homogenized sediment, they were transported directly to the laboratory. To characterize the sediment collected, we measured the organic matter content and the grain size distribution. The organic matter content was measured as AFDW (ash free dry weight) for each 0.5 mL of sediment sample, which was dried at 70 °C for 72 h and burned for 4 h at 450 °C using a muffle furnace (AAF 1100, Carbolite, UK). Overall, the organic matter content fluctuated between 0.009 ± 0.001 and 0.015 ± 0.002 grams of AFDW per gram of sediment dry weight (gAFDW/gDW) over the experimental time. In terms of grain size characterization, sediments were dominated by coarse fractions: sand and coarse-sand^30^.

In each microcosm, five leaf litter bags (3 × 3 cm, mesh size 1 mm) containing 12 leaf circles (12 mm diameter) of *Populus nigra* L. (collected just after abscission and air-dried) were placed in the subsurface sediment layers (at 7–10 cm depth) and anchored with a thin string to enable bag extraction from the surface during sampling, thus avoiding any major disruption of the surface sediment. Then, each microcosm was placed inside a larger plastic carboy (25 cm diameter, 30 cm depth) and filled with fresh filtered river water (3 L to each column) to maintain water saturation in all sediment microcosms during an acclimation period of 10 days before applying the three treatments (C, D, and DS). Water from all microcosms was renewed every two days with fresh filtered stream water to avoid nutrient depletion^58^. Water replacement was performed by emptying the carboys and filling them with new water. All microcosms were placed in incubators (SCLAB, PGA 500) under a controlled temperature and light conditions. Light conditions for the entire experiment were set at 12 h/12 h night/day, exposing the microcosms to a light irradiance (photosynthetic active radiation) of 150 μEm^−2^ s^−1^ during the day and darkness during the night. The range in temperatures applied during the experiment was set according to the average values during the spring-summer period recorded in the watershed where the sediment communities were collected (data 2005–2015 Idescat https://www.idescat.cat/pub/?id=aec&n=216&lang=en). Specifically, the day temperature range simulated the passage from late spring (25–29 °C for 6 weeks) to summer (30–31 °C for 12 weeks), while the night temperature was set 4 degrees below the maximum day value^30^.

After the first 10 days of microbial acclimation in the microcosms, the three treatments were applied. For the control treatment (C), the microcosms were maintained in saturated water conditions with a 4 cm layer of water at the top of the sediment, and the water was renewed twice a week. For the dry treatment (D), the microcosms were left to dry, the carboys were emptied, and no extra water was added for the next 150 days. For the dry-storms (DS) treatment, the microcosms were also left to dry as in the dry (D) treatment, but on days 90 and 130, 750 mL of diluted stream water (1:1, 0.2 µm filtered stream water:distilled water) was added over 30 min (simulating summer storms in that area, dataset 2005–2015 ACA – “Agència Catalana de l’Aigua”). During the experiment, the dissolved oxygen content was monitored at each sediment depth and for each treatment, while the water content was calculated as a percentage of water loss (%) obtained by the difference between the fresh and dry weights (Table S4B). From day 150, all the microcosms were rewetted and maintained under saturated conditions with filtered stream water renewed every two days. Rewetting was performed by filling the outside carboys to simulate water table resumption with the filtration of water from the bottom of the microcosms (perforated base), and at the same time, part of the water (1.5 L) was poured into the top of the microcosms to simulate precipitation and allow the wetting of the entire sediment.

### Sediment and buried leaf sampling

Surface sediment was sampled using a small syringe (2 cm diameter, 1 × 100 NORM-JECT®, Henke Sass Wolf, Germany), and re-sampling twice in the same place did not occur. Hyporheic sediment was collected with a thin spatula by opening one of the two windows created before filling the sediment containers, and then, the window was closed and hermetically sealed to prevent deep sediment oxygenation (oxygen monitoring showed that hyporheic sampling did not cause any extra oxygenation, Table S4A).

The surface (0–5 cm) and hyporheic (20–25 cm) sediment was sampled from each microcosm replicate on days 0, 52, 90 and 150 during the drought phase and after 24 h (day 151) and 7 days (day 157) during the rewetting phase (Fig. 4). On each sampling date, the treatment replicates (n = 4) were pooled and split into two subsamples: 1 mL for the analysis of microbial community diversity (bacterial, archaeal, and fungal groups) and 4 mL for sediment characterization^30^. For the buried leaves, one bag from each sediment microcosm was extracted on days 0 and 150 during the drought phase and after 24 h (day 151) and 7 days (day 157) of rewetting. A reduced frequency of sampling for the buried leaves was used since a delayed and less strong response was expected for microbes inhabiting this habitat in comparison to those in surface and hyporheic sediments and to minimize disturbance of the sediment columns (made when retrieving leaf bags). The sediment samples and leaves were immediately frozen at −20 °C for further microbial diversity analyses.

### High-throughput sequencing and sequence processing

Genomic DNA was extracted using the FastDNA™Spin Kit for Soils (MP Biomedicals, Irvine, CA), following the manufacturer’s instructions, on sediment (0.5 g) and buried leaf (4 leaf circles of 1 cm in diameter) samples. Each sample was collected at every sampling time and combined for each treatment (Fig. 4). The quantity and quality of the extracted DNA were measured spectrophotometrically (Nanodrop2000, Thermo Scientific^TM^, Waltham, USA) and further assessed through 1% agarose gel electrophoresis. DNA extracts were stored at −20 °C until sequencing analysis at the Roy J. Carver Biotechnology Center (University of Illinois, IL, US). The diversity of archaeal, bacterial and fungal communities was analysed using the primer pairs Arch 349F/Arch 806R^59^, V4_515F/V4_806R^60^, and Euk_1391F/Euk Br^61,62^ using Illumina MiSeq technology. We ran the IM-TORNADO pipeline version 2.0.3.2^63^ to generate the archaeal, bacterial and fungal OTU tables and to assign taxonomy to the corresponding OTUs (≥97% sequence similarity). For bacterial and archaeal taxonomy assignment, the Ribosomal Database Project (RDP version 10) was used^64^. For fungal taxonomy assignment, the Silva (version 128) (https://www.arb-silva.de/) database was used. Diversity analyses were performed using the IM-TORNADO-generated output BIOM table. The average sequencing depth values were 66,535 for bacteria, 19,654 for archaea and 19,889 for the total eukaryote DNA per sample. Detailed information about the proportion of all reads that were archaea, bacteria, and fungi in each treatment is reported in Supplementary Information S1.

### Statistical analyses

Rarefied OTU tables were generated by multiple rarefactions on bacterial, archaeal, and fungal BIOM tables using the *phyloseq* package by R software (R version 3.4.1). The rarefactions curves were generated (*rarefy_even_depth* function, *phyloseq* package), and then, each rarefied OTU table was set to the smallest count of reads available for any sample in the table (9212 reads per sample for V4 and archaea and 2831 reads per sample for fungi) to equalize sequencing depth samples. Alpha diversity (Shannon-Wiener and richness Chao1 indices) and the weighted UniFrac distance (measure of beta diversity) were calculated on the OTU tables (*phyloseq* package by R software, version 3.4.1). The weighted UniFrac distance matrix was estimated from the microbial phylogenetic trees (*distance* function in the *phyloseq* package, R version 3.4.1) and used as a proxy of the phylogenetic distance between the members of the communities analysed^65^. Averaged weighted UniFrac distances were estimated for all time points to describe temporal variability in phylogenetic distance^66^ for the bacterial, archaeal and fungal communities inhabiting the surface and hyporheic sediments and buried leaves. The sequencing analysis of the archaeal communities inhabiting the buried leaves was unsuccessful for most of the samples, so it was excluded from the study.

To estimate the differences depending on the three experimental factors (treatment, TR; time, TI; and habitats, HAB), PERMANOVA analyses based on 9999 permutation were run for the diversity indices and phylogenetic distance, addressing the problem of potential autocorrelation (PRIMER 6 version 6.1.11 & PERMANOVA + version 1.0.1). Because significant differences were observed between habitats (as surface and hyporheic sediments and buried leaves), further PERMANOVA analyses specific for each habitat were assessed (treatment, TR and time, TI as factors; PRIMER 6 version 6.1.11 & PERMANOVA + version 1.0.1). Furthermore, given the potential problem related to combining samples, the diversity results were further analysed by repeated measures to address the potential pseudo-replication (method: scaled identity; results shown in Supplementary Information S2) (IBM SPSS STATISTICS 25 software (SPSS Inc., USA)). For the community composition analysis, principal coordinates analysis (PCO) provided an ordination of the bacterial, archaeal and fungal communities in the different habitats and subjected these communities to the different treatments in a factorial map based on the scores of the first two principal components. The abundance matrices used for the ordination analyses were previously fourth-root transformed, and distance matrices were obtained based on Bray-Curtis dissimilarity. For the PCO analyses, the permutation multivariate analysis of variance PERMANOVA analyses were combined to determine the eventual community composition differences between treatments (C, D, and DS), types of habitats (sediment surface, hyporheic, and buried leaves) and their interaction, considering 9999 permutations (PRIMER 6 version 6.1.11 & PERMANOVA + version 1.0.1). The shifts in the archaeal, bacterial, and fungal class abundances (with relative abundances > 5%) between the C, D, and DS treatments were tested by one-way ANOVA (dixed factor = treatment (C, D, DS) and represented by box plots including all the times (from 0 to 157 days)) using IBM SPSS STATISTICS 25 software (SPSS Inc., USA). Finally, the percentage of shared OTUs (%) between treatments and for each microbial group (bacteria, archaea, and fungi) was detected by the average value obtained by a Venn diagram study (data not shown) using the Venn Diagram library (Venny 2.1, Oliveros (2007–2015) http://bioinfogp.cnb.csic.es/tools/venny/index.html).

## Supplementary information

Supplementary materials

## Data Availability

Sequencing data were deposited in the NCBI (National Center for Biotechnology Information) under SRA accession (sequence read archive) number: PRJNA507856.
