# Supplementary material for: Distinct responses from bacterial, archaeal and fungal streambed communities to severe hydrological disturbances
Source: Sci Rep. 2019 Sep 18;9:13506. doi: 10.1038/s41598-019-49832-4 (PMC6751160; doi:10.1038/s41598-019-49832-4)
Supplement: Supplementary file 1 — Supplementary materials [file 41598_2019_49832_MOESM1_ESM.pdf]

Supplementary materials for:

Distinct responses from bacterial, archaeal and fungal streambed communities to severe hydrological disturbances

Gionchetta G. [1,*], Romaní A.M. [1,+], Oliva F. [2], Artigas J. [3,+]

[1]GRECO, Institute of Aquatic Ecology, University of Girona, 17003 Girona, Spain
[2]Department of Genetics, Microbiology and Statistics, University of Barcelona, Barcelona, Spain
[3]Université Clermont Auvergne, CNRS, Laboratoire Microorganismes: Génome et Environnement, F-63000 Clermont-Ferrand, France
[*]Corresponding author e-mail address: giulia.gionchetta@udg.edu
[+] These authors contributed equally to this work

Table of contents:

Fig. S1.............................Pages 2-3

Fig. S2.............................Page 4

Fig. S3.............................Page 5

Fig. S4.............................Page 6

Fig. S5.............................Page 7-8

Fig. S6.............................Page 9

Table S1..........................Page 10

Table S2..........................Page 11

Table S3..........................Page 12

Table S4..........................Page 13

Table S5..........................Page 14

Supplementary information S1 ....Page 14

Supplementary information S2 ....Page 15

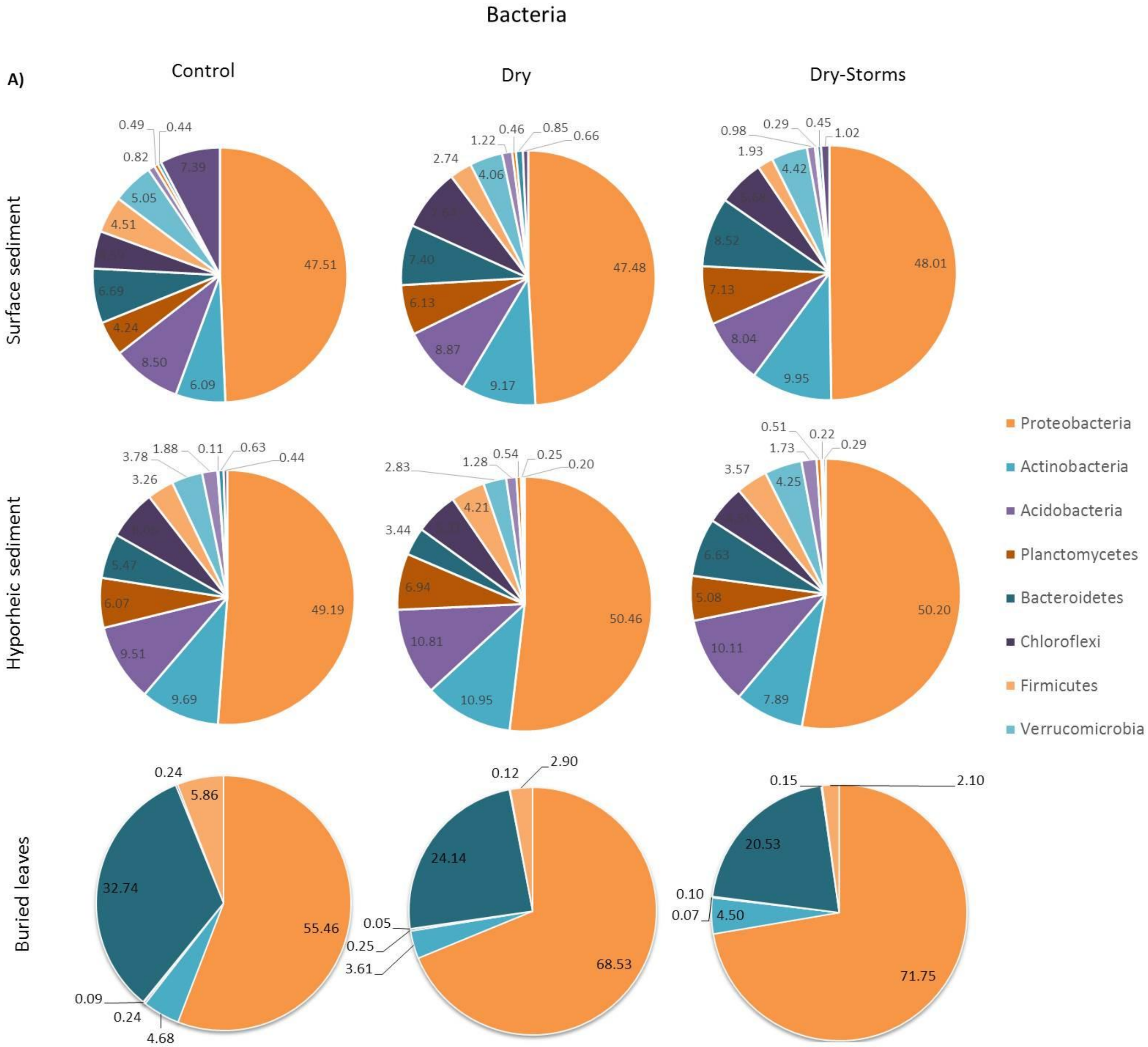
Bacteria
A)
Control
Dry
Dry-Storms
Surface sediment
Hyporheic sediment
Buried leaves
0.49
0.44
0.82
7.39
5.05
4.51
47.51
6.69
4.24
8.50
6.09
2.74
1.22
0.46
0.85
0.66
4.06
7.40
6.13
47.48
8.87
9.17
0.98
0.29
0.45
1.02
1.93
4.42
8.52
7.13
48.01
8.04
9.95
3.78
1.88
0.11
0.63
0.44
3.26
5.47
6.07
49.19
9.51
9.69
2.83
1.28
0.54
0.25
0.20
4.21
3.44
6.94
50.46
10.81
10.95
0.51
0.22
0.29
1.73
3.57
4.25
6.63
5.08
50.20
10.11
7.89
Proteobacteria
Actinobacteria
Acidobacteria
Planctomycetes
Bacteroidetes
Chloroflexi
Firmicutes
Verrucomicrobia
0.24
5.86
32.74
55.46
0.09
0.24
4.68
0.12
2.90
24.14
0.05
0.25
3.61
68.53
0.15
2.10
20.53
0.10
0.07
4.50
71.75

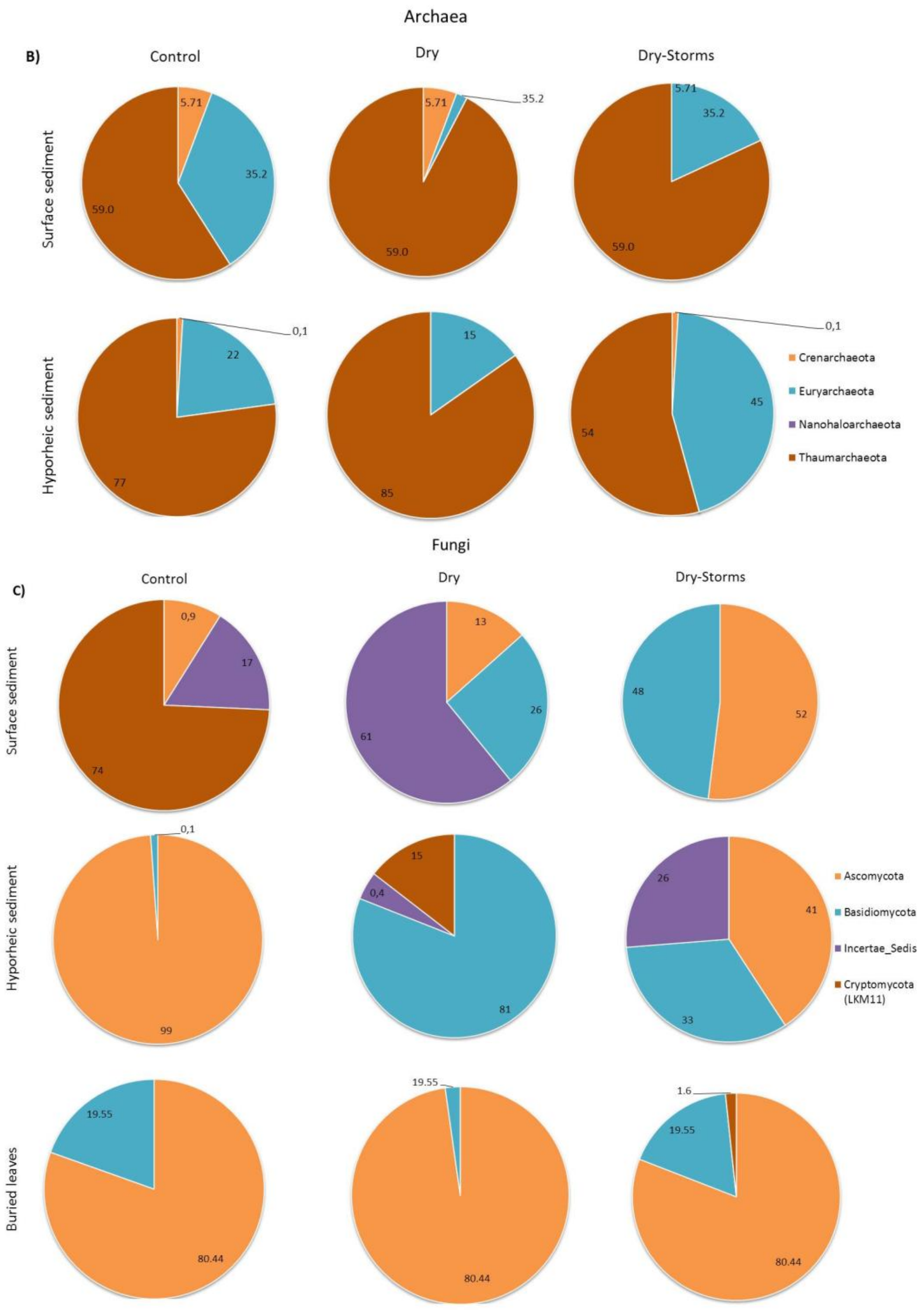

Fig. S1 Cake plots representing initial microbial composition (time 0) for Control, Dry and Dry-Storm treatments in surface and hyporheic sediments and buried leaves for: **A)** bacteria, **B)** archaea, **C)** fungi. These results are associated to those presented in the Table S1.

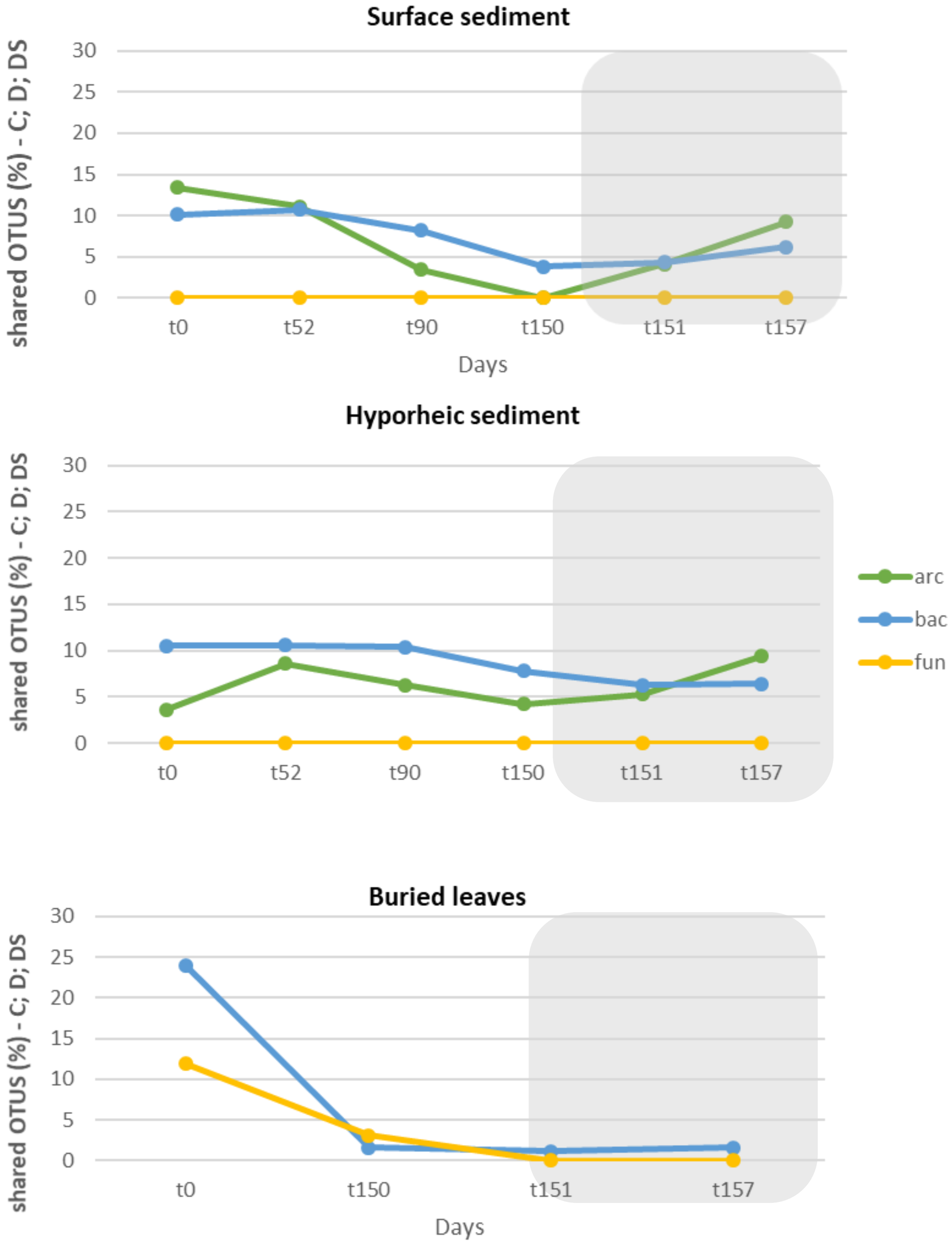

Fig. S2 Percentage of shared OTUs between the three treatments (C, Control; D, Dry; DS, Dry-Storm) reported during the experimental time (0, 52, 90, 150, 157 days), from each community (bac., bacteria; arc., archaea and fun., fungi) inhabiting each habitat (surface and hyporheic sediment and buried leaves). The shaded area indicate rewetting phase.

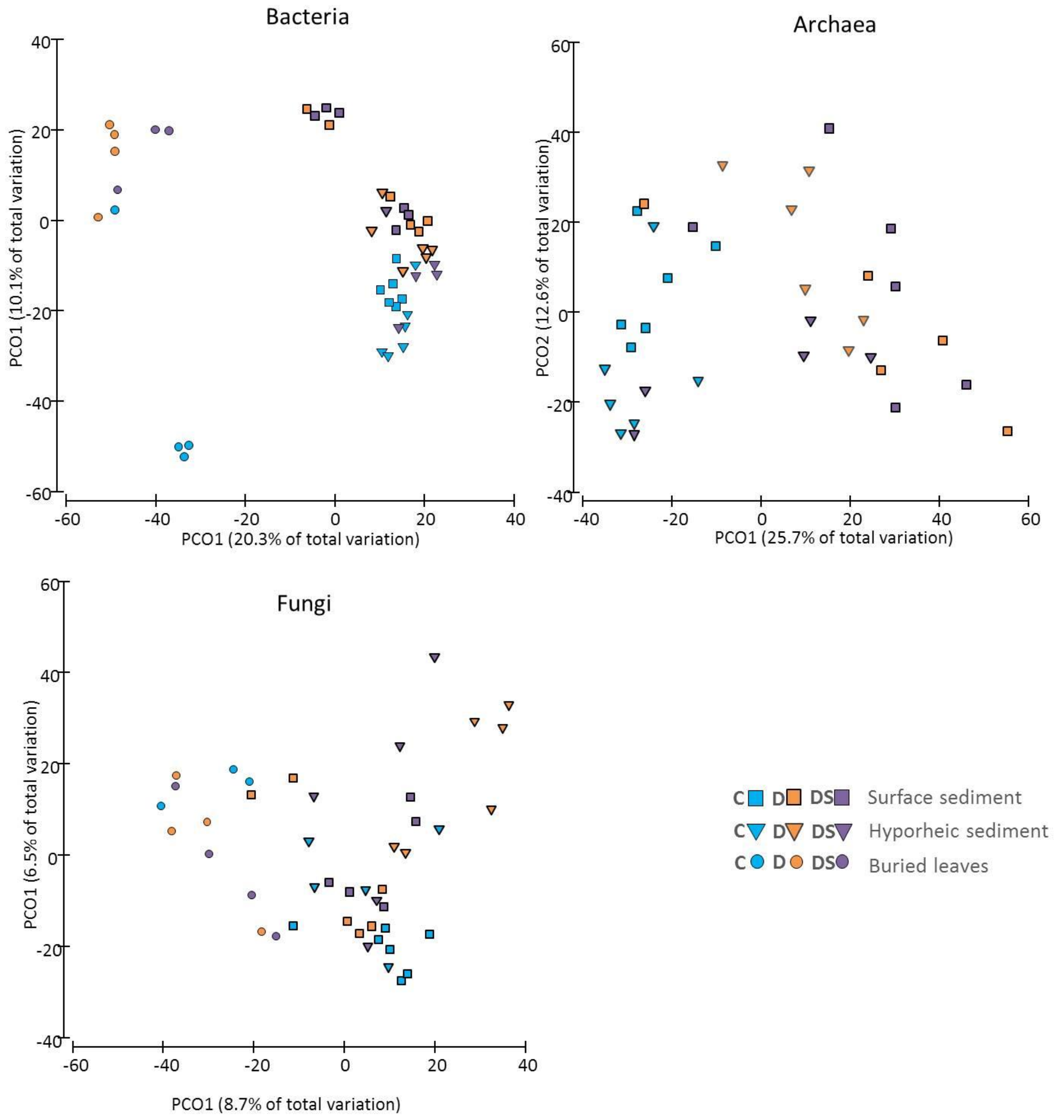

Fig.S3 PCO from bacterial, archaeal and fungal OTUs, inhabiting the three habitats, indicated as: surface sediment; hyporheic sediment; buried leaves. The colour pattern is indicated in the legend for the three treatments: C, Control; D, Dry; DS, Dry-Storm.

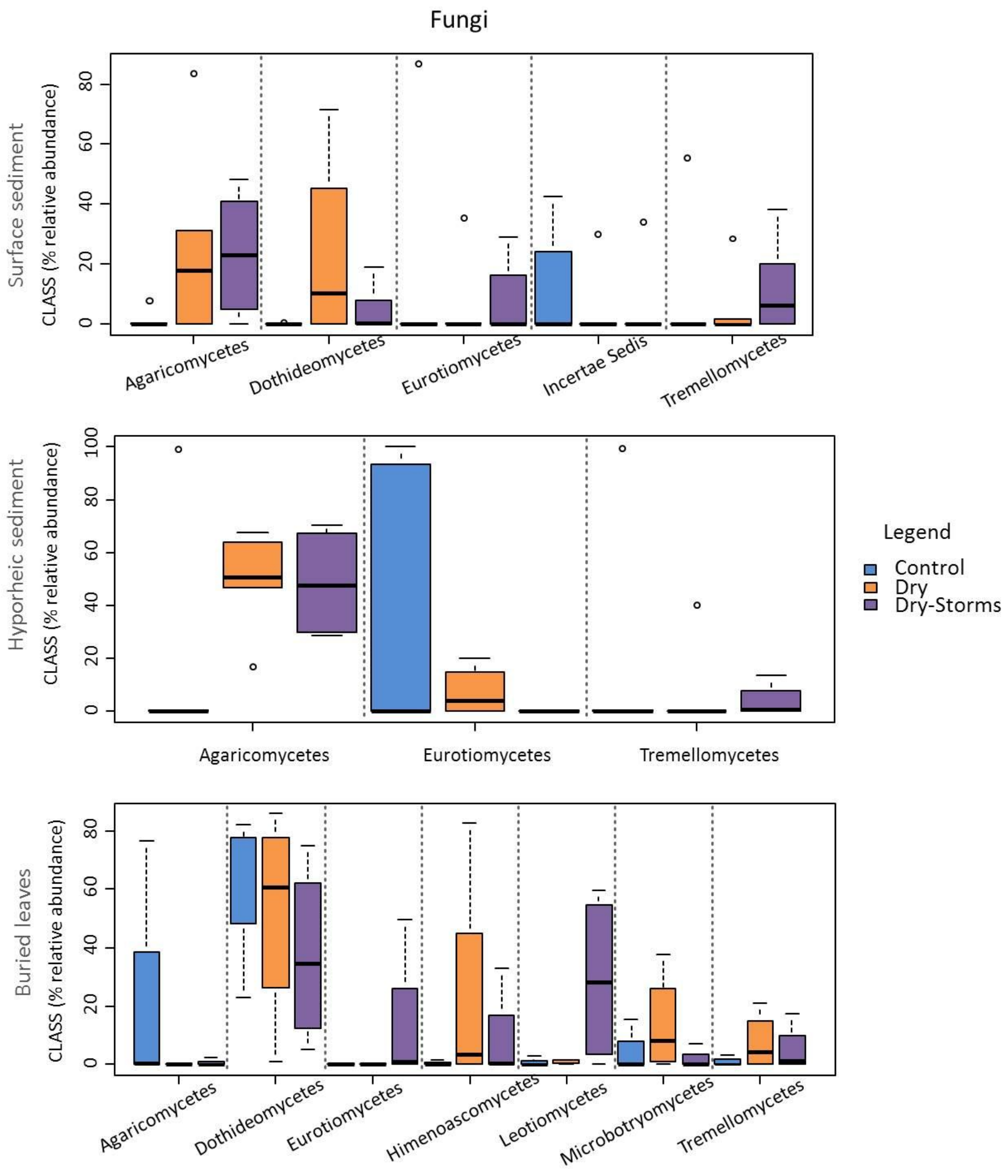

Fig.S4 Boxplots for fungal classes variability (% of relative abundance) considering the whole experimental period. The three habitats are indicated as: surface sediment; hyporheic sediment; buried leaves. The colour pattern is indicated in the legend for the three treatments: C, Control; D, Dry; DS, Dry-Storm. Only the classes presenting relative abundances >5% have been considered.

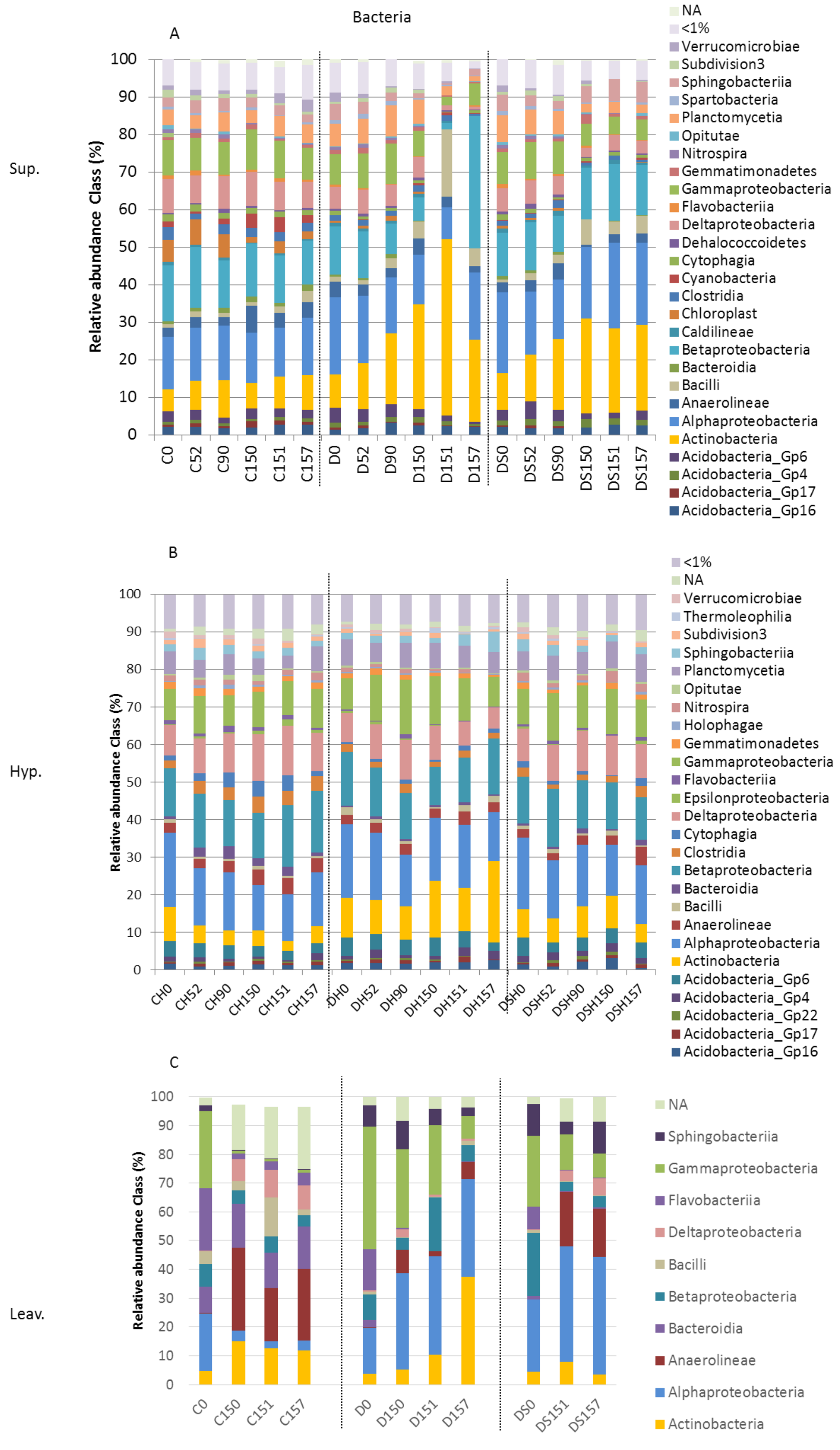

Fig.S5A-B-C Histogram of bacterial class composition (% of relative abundance) inhabiting surface (A) and hyporheic (B) sediment and buried leaves (C). In the *x-axis* the three treatments are indicated (C, D, DS) for each sampling time (day 0, 52, 90, 150, 151, 157).

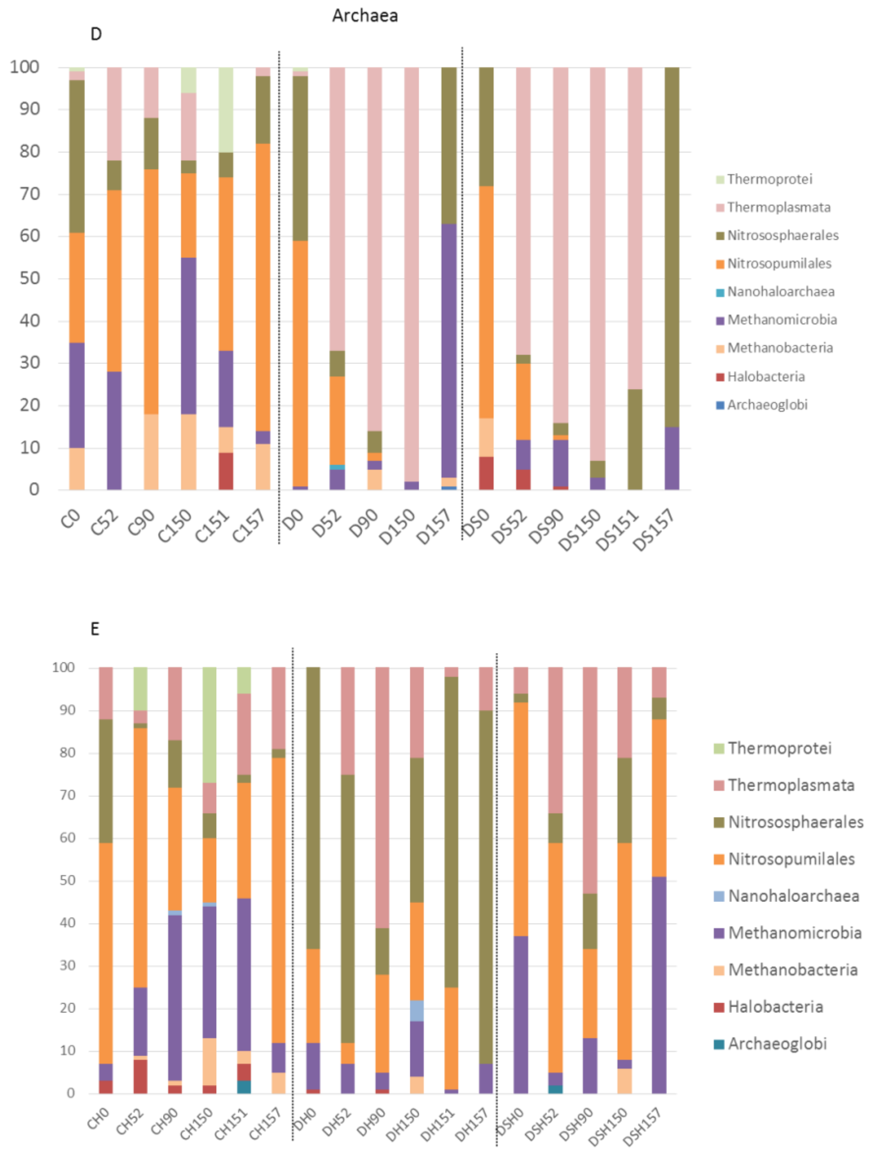

Fig.S5D-E Histogram of archaeal class composition (% of relative abundance) inhabiting surface (D) and hyporheic (E) sediment. In the *x-axis* the three treatments are indicated (C, D, DS) for each sampling time (day 0, 52, 90, 150, 151, 157).

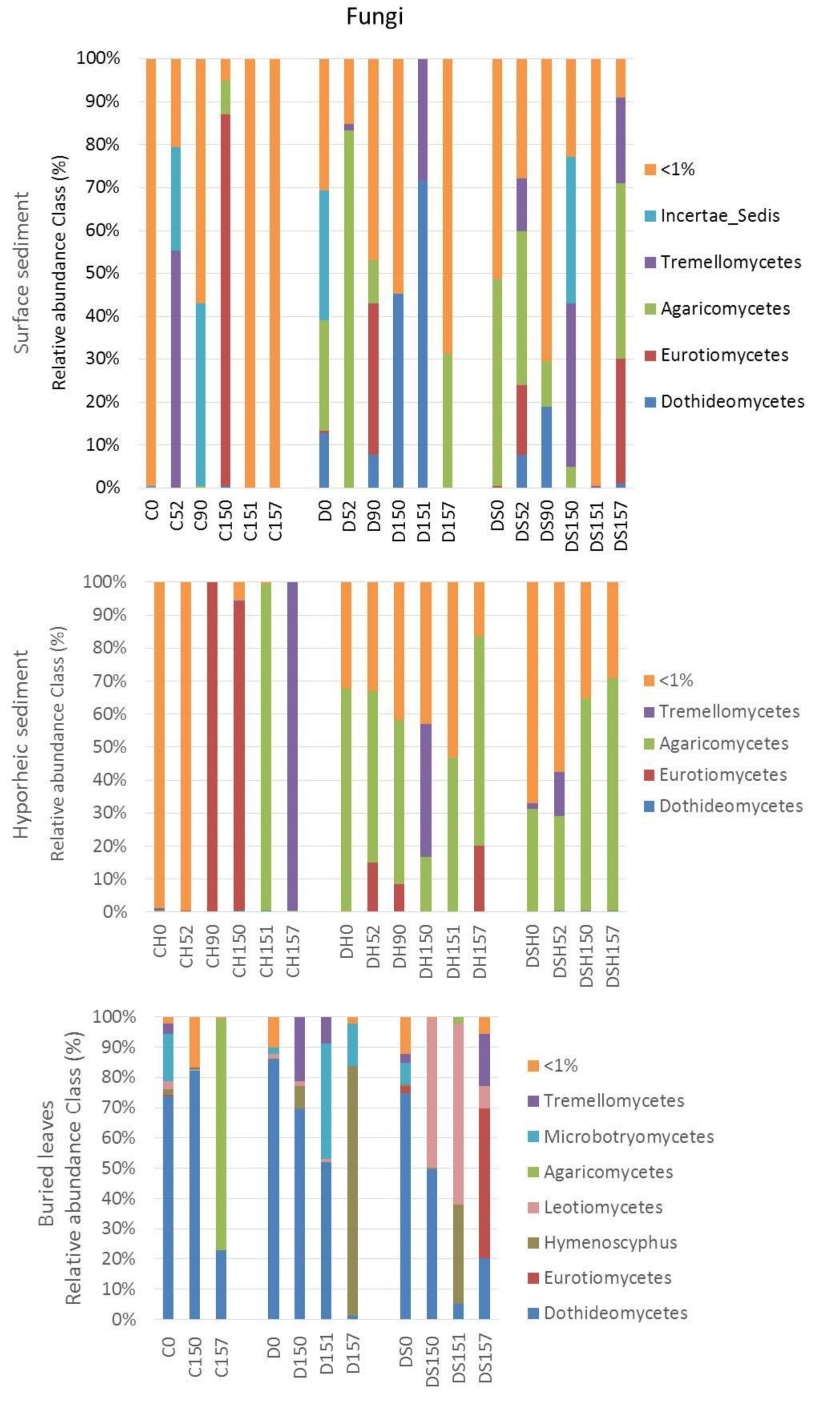

Fig.S6 Histograms of fungal class composition (% of relative abundance) for the three habitats studied, indicated as: surface sediment; hyporheic sediment; buried leaves.In the *x-axis* the three treatments are indicated (C, D, DS) for each sampling time (day 0, 52, 90, 150, 151, 157).

Table S1. Results (p-values) from the PERMANOVA analyses applied over the initial communities (referred to Figure S1). The acronyms SUR and HYP state for Surface and Hyporheic sediment, respectively. The letters C, D and DS state for the three treatments Control, Dry and Dry-Storms respectively.

	BACTERIA			ARCHAEA		FUNGI		
	SUR	HYP	LEAVES	SUR	HYP	SUR	HYP	LEAVES
C, D	0.329	0.662	0.671	0.670	0.332	0.670	0.664	0.663
C, DS	0.335	1.000	0.672	0.669	0.336	0.333	1.000	0.669
D, DS	1.000	0.343	1.000	0.671	0.327	0.666	1.000	0.672

Table S2. Richness (S) and Shannon diversity (H), based on number of OTUs of bacterial, archaeal and fungal assemblages at four dry-phase times (t0, t52, t90, t150) and at two rewetting-phase times (t151, t157), for three different habitats (SUR, surface sediment; HYP, hyporheic sediment; LEAVES, buried leaves) and three treatments (C, Control; D, Dry; DS, Dry-Storms).

			t0		t52		t90		t150		t151		t157	
	**Habitat**	**Treat.**	**S**	**H**	**S**	**H**	**S**	**H**	**S**	**H**	**S**	**H**	**S**	**H**
**Bacteria**	SUR	C	3107.1	6.98	3055.2	7.02	2094.1	6.64	2391.5	6.72	2282.4	6.73	2127.1	6.63
		D	2880.8	7.03	3388.2	7.22	2897.5	7.03	2335.3	6.63	1140.7	4.58	1585.7	5.18
		DS	3317.1	7.15	3886.3	7.35	2931.6	7.00	1807.8	6.08	1962.6	6.18	2348.6	6.46
	HYP	C	3092.7	7.09	3218.8	7.18	3271.0	7.18	2697.5	7.00	2738.1	6.96	2801.2	7.06
		D	2711.5	6.90	3538.4	7.26	3577.8	7.27	3204.7	7.17	1938.1	6.53	2150.8	6.40
		DS	2751.6	6.91	2912.5	7.09	3272.8	7.20	2071.0	6.65	2201.1	6.75	2201.1	6.75
	LEAVES	C	925.1	4.55	na	na	na	na	1041.2	4.50	1125.5	4.80	998.7	4.81
		D	766.0	4.40	na	na	na	na	672.1	4.42	856.0	4.45	489.2	4.35
		DS	964.6	4.67	na	na	na	na	1064.4	4.77	1164.3	4.87	910.4	4.96
**Archaea**	SUR	C	33.0	2.54	17.0	1.89	11.0	1.92	12.0	2.14	23.5	2.27	21.0	1.40
		D	16.0	1.77	16.5	2.42	19.0	1.99	9.0	1.71	na	na	5.0	0.91
		DS	20.5	1.83	20.0	2.50	16.0	1.84	6.0	0.59	6.0	1.08	12.0	2.01
	HYP	C	18.5	2.10	25.5	2.28	47.5	2.89	29.5	2.94	33.0	2.72	39.0	2.35
		D	14.7	2.09	20.5	1.55	22.0	2.39	16.5	2.55	12.0	1.90	10.0	1.82
		DS	11.0	1.49	17.5	2.31	22.0	2.68	22.2	2.10	na	na	17.0	2.16
**Fungi**	SUR	C	4.0	0.76	6.0	1.55	8.5	1.55	8.5	0.60	6.0	1.22	3.0	0.58
		D	7.0	1.57	5.0	0.96	14.0	2.30	6.0	1.12	3.0	0.63	3.0	0.74
		DS	7.0	1.39	16.2	2.45	9.0	1.63	5.0	1.30	5.0	1.35	11.0	1.76
	HYP	C	8.0	0.76	4.0	1.55	1.0	1.55	9.0	0.60	4.5	1.22	2.0	0.58
		D	5.0	1.57	18.5	0.96	7.0	2.30	5.0	1.12	6.0	0.63	7.0	0.74
		DS	6.0	1.39	12.5	2.45	na	1.63	3.0	1.30	na	1.35	3.0	1.76
	LEAVES	C	31.5	1.76	na	na	na	na	25.0	1.02	na	na	6.0	0.68
		D	21.7	1.53	na	na	na	na	46.0	2.14	7.0	1.45	5.0	0.58
		DS	19.3	1.68	na	na	na	na	22.0	1.84	6.0	1.06	17.0	1.75

Table S3. *p*-values from PERMANOVA analyses from complete PCO (Fig. S3). Differences between *Treatments* (C, D, and DS), type of *Habitats* (surface and hyporheic sediment, buried leaves) and the interaction *Treatment* x *Habitats* were assessed for each microbial community (bacteria, archaea, fungi). p-values indicating significant difference ($p<0.05$) are indicated in boldface while p-values at the limit of significance ($p<0.1$) are indicated in italic.

	**Bacteria**	**Archaea**	**Fungi**
Treatment	**<0.001**	**<0.001**	0.516
Habitats	**<0.001**	**0.025**	**<0.001**
Treatment x Habitats	**0.003**	*0.080*	**0.047**

Table S4. A) Dissolved oxygen (DO) in the sediment monitored during the experimental period and the average (±SD) for each treatment and habitat; **B)** Water sediment content (%±SD) assessed at each sampling time. The habitats are indicated with the abbreviations as SUP: surface sediment and HYP: hyporheic sediment whereas the three treatments are stated as C: Control, D: Dry, DS: Dry-Storms.

A

Sampling date (day)		DO (mg/l)										
		19-feb	*03-mar*	*17-mar*	*05-apr*	*19-apr*	*04-may*	*24-may*	*25-jun*	*15-jul*	**DO average**	**SD**
SUP	C	3.90	5.75	6.08	5.42	5.51	1.15	1.94	4.03	2.87	4.07	1.68
	D	7.42	8.09	8.87	8.70	8.90	8.74	8.92	8.52	8.68	8.54	0.46
	DS	7.50	8.29	8.81	8.76	8.81	9.67	8.97	8.69	8.86	8.71	0.55
HYP	C	0.08	0.08	0.03	0.06	0.03	0.17	0.04	0.07	0.07	0.07	0.04
	D	7.08	7.78	9.06	8.80	8.86	8.96	8.80	8.48	8.68	8.50	0.61
	DS	4.32	8.32	8.73	8.65	9.02	9.46	9.20	8.24	8.80	8.31	1.46

B

		Water content average (%) ± SD					
Sampling time (day)		*0*	*52*	*90*	*150*	*151*	*157*
SUP	C	17.27±2.19	29.5±0.54	28.48±0.43	29.25±1.29	21.96±1.33	23.86±4.43
	D	15.58±1.55	0.83±0.54	0.34±0.37	0.44±0.91	13.68±1.33	22.71±4.43
	DS	16.57±1.26	1.14±0.54	0.36±0.37	2.73±0.91	12.63±1.33	21.58±4.43
HYP	C	43.29±1.30	27.74±1.51	26.61±0.90	27.45±1.21	20.57±2.11	23.73±2.68
	D	44.71±1.06	4.04±1.06	2.82±0.64	0.65±1.05	18.46±2.11	22.06±2.68
	DS	43.43±1.3	4.55±1.06	2.86±0.64	5.48±1.05	20.01±2.11	20.31±2.68

Table S5. Physicochemical parameters in stream water monitored during the experimental period and further checked with the ACA dataset ("Agència Catalana de l'Aigua" dataset January 2016 - August 2016). During the study period the stream was in base flow conditions and no storms occurred.

	**Mean value**	**Unit**
**N-NO2**	0.05 ± 0.12	mg/L
**N-NO3**	0.14 ± 0.12	mg/L
**P-PO4**	0.83 ± 0.12	mg/L
**DOC**	10.43 ± 1.38	mg/L
**Water Tº (range)**	7 - 15.6	ºC
**Dissolved Oxygen**	10.18 ± 3.58	mg/L
**Conductivity**	488 ± 41.39	µS/cm
**pH**	8.54 ± 0.06	

Supp. Info. S1. Detailed information about the total (TOT) reads of prokaryotes (PROK) and eukaryotes (EUK) and the contribution in percentage (%) for the three communities: archaea (Arc), bacteria (Bac) and fungi (Fun) in each treatment (Control, Dry and Dry-Storms). The information is presented for each microhabitat considered: surface sediment, hyporheic sediment and buried leaves. The "na" identifies not available data.

Surface sediment

PROK	*Control*	*Dry*	*Dry-Storms*
TOT	70303	64537	67700
**Arc (%)**	5.58	14.54	10.18
**Bac (%)**	94.42	85.46	89.82
PROK+EUK	*Control*	*Dry*	*Dry-Storms*
TOT	71355	67071	69545
**Arc (%)**	5.50	13.99	9.91
**Bac (%)**	93.03	82.23	87.44
**Fun (%)**	1.47	3.78	2.65

Hyporheic sediment

PROK	*Control*	*Dry*	*Dry-Storms*
TOT	60985	60517	68147
**Arc (%)**	11.90	10.78	12.12
**Bac (%)**	88.10	89.22	87.88
PROK+EUK	*Control*	*Dry*	*Dry-Storms*
TOT	62795	62743	70074
**Arc (%)**	11.56	10.40	11.79
**Bac (%)**	85.56	86.05	85.46
**Fun (%)**	2.88	3.55	2.75

Buried leaves

PROK+EUK	*Control*	*Dry*	*Dry-Storms*
TOT	73299	55493	61083
**Arc (%)**	na	na	na
**Bac (%)**	90.22	89.35	91.24
**Fun (%)**	9.78	10.65	8.76

Suppl. Info. S2. Results from the repeated measures for Shannon-Wiener diversity (H) and Richness (S) indices and for the Unifrac distance (proxy of phylogenetic distance). The acronyms TR, TI and HAB indicate Treatments, Time and Habitats (surface and hyporheic zone), respectively.

**BACTERIA - H index**			**ARCHAEA - H index**			**FUNGI - H index**		
Origin	F	Sign.	Origin	F	Sign.	Origin	F	Sign.
Intercept	18,598,808	,000	Intercept	1,074,519	,000	Intercept	158,212	,000
TR	3,732	*,062*	TR	5,710	**,029**	TR	4,758	**,044**
TI	9,101	**,002**	TI	1,735	,233	TI	2,949	*,084*
HAB	14,188	**,004**	HAB	8,695	**,018**	HAB	5,242	**,051**
TR * TI	3,105	**,044**	TR * TI	2,117	,150	TR * TI	,623	,763
TR * HAB	1,971	,190	TR * HAB	,250	,785	TR * HAB	3,699	*,073*
TI * HAB	2,507	,101	TI * HAB	3,288	*,066*	TI * HAB	1,633	,256

**BACTERIA – S index**			**ARCHAEA – S index**			**FUNGI – S index**		
Origin	F	Sign.	Origin	F	Sign.	Origin	F	Sign.
Intercept	7,810,217	,000	Intercept	248,703	,000	Intercept	103,463	,000
TR	1,657	,239	TR	11,250	**,005**	TR	,933	,432
TI	48,502	**,000**	TI	,968	,490	TI	2,400	,130
HAB	19,662	**,001**	DE	5,431	**,048**	HAB	1,796	,217
TR * TI	7,657	**,002**	TR * TI	,754	,669	TR * TI	1,518	,283
TR * HAB	11,459	**,003**	TR * DE	1,892	,212	TR * HAB	2,607	,134
TI * HAB	7,602	**,003**	TI * DE	2,604	,110	TI * HAB	1,548	,277

**BACTERIA Unifrac**			**ARCHAEA Unifrac**			**FUNGI Unifrac**		
Origin	F	Sign.	Origin	F	Sign.	Origin	F	Sign.
Intercept	2,523,566	,000	Intercept	775,760	,000	Intercept	520,677	,000
TR	14,794	**,001**	TR	,894	,446	TR	1,578	,264
TI	1,553	,259	TI	1,722	,235	TI	,285	,908
HAB	21,557	**,001**	HAB	11,416	**,010**	HAB	1,223	,301
TR * TI	,935	,541	TR * TI	1,127	,441	TR * TI	,600	,779
TR * HAB	14,658	**,001**	TR * HAB	3,875	*,067*	TR * HAB	5,205	**,036**
TI * HAB	,220	,946	TI * HAB	2,000	,183	TI * HAB	,492	,774

**BACTERIA - H index**		**LEAVES**	**FUNGI - H index**		**LEAVES**
Origin	F	Sign.	Origin	F	Sign.
Intercept	26,234,067	,000	Intercept	80,404	,000
TR	17,734	**,003**	TR	,891	,467
TI	2,529	,154	TI	1,452	,333

**BACTERIA – S index**		**LEAVES**	**FUNGI – S index**		**LEAVES**
Origin	F	Sign.	Origin	F	Sign.
Intercept	1,675,366	,000	Intercept	33,297	,002
TR	23,995	**,001**	TR	,165	,853
TI	5,374	**,039**	TI	3,766	*,094*
